# Effectiveness of preimplantation genetic testing in sickle cell disease: insights from a single-center experience

**DOI:** 10.1007/s10815-026-03809-1

**Published:** 2026-02-05

**Authors:** A. Aganahi, F. Souare, A. Mayeur, H. Thomas, S. Monnot, A. Benachi, L. Joseph, A. Habibi, N. Frydman, M. Grynberg, J. Steffann, C. Sonigo

**Affiliations:** 1 Service de Médecine de La Reproduction Et Préservation de La Fertilité, AP-HP, Université Paris-Saclay, Hôpital Antoine Beclère, 157 Avenue de La Porte Trivaux, 92140 Clamart, France; 2 Service d’Histologie – Embryologie- CECOS, AP-HP, Université Paris-Saclay, Hôpital Antoine Beclère, Clamart, France; 3 Service de Médecine Génomique Des Maladies Rares, AP-HP, Hôpital Necker-Enfants Malades, Paris, France; 4 Service de Gynécologie – Obstétrique, AP-HP, Université Paris Saclay, Hôpital Antoine Béclère, Clamart, France; 5 AP-HP, Hôpital Universitaire Necker-Enfants Malades, Service Hémaphérèse Thérapeutique, Paris, France; 6 Sickle Cell Referral Center, Unité Des Maladies Génétiques du Globule Rouge, Henri MONDOR University Hospital, AP-HP, INSERM, U955, DHU A-TVB, Equipe Transfusion Et Maladies du Globule Rouge, IMRB, Creteil, France

**Keywords:** Sickle cell disease, PGT-M, Preimplantation genetic diagnosis, Ovarian stimulation

## Abstract

**Purpose:**

Sickle-cell disease (SCD) is a severe autosomal recessive disorder. At-risk couples may prevent transmission either through prenatal diagnosis with possible termination of pregnancy or preimplantation genetic testing for monogenic disease (PGT-M). Data on PGT-M outcomes in this population remain scarce.

**Methods:**

We conducted a monocentric retrospective study (2006–2021). To assess ovarian response to stimulation, each PGT-M cycle for SCD was matched with two control cycles.

**Results:**

Sixty couples underwent at least one ovarian stimulation for PGT procedure for SCD. Eight couples (13.3%) had one affected partner (S/S or S/C) and one carrier (A/S), while 52 couples (86.7%) were both carriers (A/S). Thirty-five couples (58.3%) already had an affected child, and 17 couples (28.3%) requested PGT-M with HLA typing. Median female age at first attempt was 33 years. Overall, 19 couples (31.7%) achieved at least one live birth following fresh or frozen embryo transfer. Among the 17 couples requesting HLA typing, three HLA-matched births (15.7%) and one unmatched healthy birth were achieved. None of the five women affected by SCD achieved a live birth. Ovarian response did not differ significantly between women with sickle cell trait and the controls.

**Conclusion:**

PGT-M is as a viable option for obtaining healthy offspring. These results bolster the argument that PGT-M serves as an alternative to prenatal diagnosis for eligible couples. Our study aims to assist geneticists, gynecologists, and hematologists in providing the necessary guidance before embarking couples on this long and often challenging journey.

**Supplementary Information:**

The online version contains supplementary material available at 10.1007/s10815-026-03809-1.

## Introduction

Sickle cell disease (SCD) is one of the most common genetic disorders in France and worldwide, affecting more than 5 million individuals, with over 500,000 affected newborns each year [1]. Advances in medical care have significantly improved survival, with life expectancy now exceeding 40 years in many countries. Consequently, an increasing number of individuals with SCD or sickle cell trait reach reproductive age and express a desire to conceive [2, 3]. However, in women with SCD, pregnancy remains a high-risk condition due to an increased incidence of maternal and fetal complications associated with the disease.

SCD is an autosomal recessive disorder caused by pathogenic variants in the *HBB* gene, leading to the production of abnormal hemoglobin. The disease mainly includes homozygous hemoglobin S (SS) and compound heterozygous forms such as hemoglobin SC (SC), which are associated with variable clinical severity. In contrast, heterozygous carriers (AS), commonly referred to as having *sickle cell trait*, are usually asymptomatic but can transmit the disease. Clinical expression among affected individuals is highly heterogeneous; approximately 20% of patients present with particularly severe forms of SCD, characterized by a high burden of complications and healthcare utilization, as reported in a large national cohort study [4]. Vaso-occlusive crises (VOC), defined as acute episodes of severe pain caused by microvascular obstruction, and Acute-chest syndrome (ACS) represent the most frequent acute complication and a major cause of hospitalization in patients with SS and SC phenotypes.

Despite therapeutic advances, management of SCD remains largely supportive, relying primarily on transfusion programs and hydroxyurea to reduce the frequency of severe complications [5], with persistent concerns regarding their use before conception or during pregnancy, despite reassuring data [6, 7]. For selected patients, hematopoietic stem cell transplantation (HSCT) remains the only curative treatment when an HLA-matched sibling donor is available. However, HSCT—particularly its conditioning regimens—is associated with significant gonadotoxicity, including premature ovarian insufficiency in women and azoospermia in men [8]. These limitations highlight the importance of reproductive strategies that both prevent disease transmission and may facilitate access to curative treatment.

In this context, couples at risk of transmitting SCD may benefit from preconception counseling, including prenatal diagnosis or preimplantation genetic testing for monogenic diseases (PGT-M). PGT-M relies on in vitro fertilization (IVF) to select and transfer unaffected embryos, thereby avoiding invasive prenatal diagnosis and potential pregnancy termination. In France, since the Early 2000s, PGT-M for SCD may be combined with HLA typing, allowing the selection of embryos that are both unaffected and HLA-matched with an affected sibling. This strategy offers families the opportunity to prevent SCD transmission while enabling the birth of an HLA-matched sibling who may serve as a donor for HSCT, although the proportion of transferable embryos remains limited in autosomal recessive condition.

Nevertheless, the PGT-M process may be long, emotionally demanding, and without guarantee of success. Moreover, data regarding IVF outcomes, ovarian response to controlled ovarian hyperstimulation (COH), and PGT-M success rates in couples at risk of SCD transmission remain scarce [9–11]. In particular, it is unclear whether these outcomes differ between individuals with sickle cell trait and those with sickle cell disease. The present retrospective single-center study reports a large cohort of couples undergoing PGT-M for SCD. The primary objective was to evaluate IVF and PGT-M outcomes to improve pre-treatment counseling, with particular attention to couples requesting combined PGT-M and HLA typing. A secondary objective was to assess ovarian response to COH and PGT-M outcomes per cycle in this population.

## Material and methods

The present study is a monocentric retrospective observational study. All couples who started a PGT-M procedure for the risk of transmitting SCD to their offspring between 2006 and 2021 at our PGT center were included. In order to evaluate the response to ovarian stimulation in woman at risk of transmitting SCD, we conducted a case–control study.

### Patients

#### Couple at risk of SCD transmission

All men and women included in the study were carriers of at least one mutation of the *HBB* gene. All couples had started at least one ovarian stimulation cycle for the PGT-M. Before inclusion in the PGT-M program, feasibility of the procedure was assessed through evaluation of ovarian reserve in women and sperm analysis in men. Taking into account the 18-month waiting time for PGT at our center, only women under 37 years of age at the time for PGT demand and with serum anti-Müllerian hormone (AMH) level above 1 ng/ml and/or an antral follicle count (AFC) determined by ultrasound AFC above 10 follicles were accepted in the PGT-M program. AMH and AFC were controlled 6 months before the first PGT-M procedure.

#### Control group

Each COH cycle of the SCD group was matched with two control COH cycles (ratio 1:2) corresponding to PGT-SR for couples where the man partner carried structural rearrangement. Matching criteria were (i) woman’s age at stimulation ± 2 years; (ii) same COS protocol; (iii) starting FSH dose ± 50 IU; (iv) AFC on the first day of COH ± 5 follicles; and (v) year of the first PGT procedure ± 3 years.

### Controlled ovarian stimulation protocols and PGT procedure

COH was achieved using an antagonist protocol or a long agonist as previously described [11–13]. Initial dose of gonadotropins was individualized on patient AFC, AMH level, body mass index and previous ovarian hyperstimulation. When at least three preovulatory follicles (16–22 mm in diameter) were observed, ovulation was triggered with hCG and/or triptorelin 0.2 mg.

For SCD women, prior to oocyte retrieval, preventive measures, as thrombo-embolic prophylaxis using anticoagulation and a dedicated hydration protocol, were discussed in advance within a multidisciplinary team involving sickle cell specialists and anesthesiologists to ensure optimal safety and individualized care to prevent the thrombo-embolic risk and the risk of VOC or acute ACS.

Cumulus-oocyte complexes (COCs) where retrieved 36–38 h after trigger by transvaginal ultrasound guided aspiration under local or general anesthesia. COCs recovered were denuded and the metaphase II oocytes (MII) were inseminated by ICSI. If possible, embryo biopsy was performed at the cleavage or blastocyst stage using a laser procedure as previously described [12, 13]. Genetic analysis was performed by single-cell multiplex PCR for short tandem repeat-based haplotyping and was followed by enzymatic digestion for the detection of the *HBB* gene mutation. Embryos homozygous for the mutant allele were diagnosed as affected and were not transferred. When available, one or two unaffected embryos were transferred into the uterus on day 4 or 5 after ICSI. Women affected by SCD underwent single embryo transfers only, due to medical considerations. Supernumerary and good quality unaffected embryos were cryopreserved either by slow freezing or vitrification, according to the time of the PGT-M procedure. Blood test for pregnancy was performed 10–15 days after embryo transfer and if positive, an ultrasound was scheduled 4–6 weeks later to confirm the pregnancy localization and evolution.

### Study variables

The collected data included the following: couple characteristics at the start of the first PGT procedure (age, body mass index (BMI), pregnancy history and outcomes, sickle cell status of both partners (heterozygous or homozygous), woman’s ovarian reserve assessment (AMH, AFC)); characteristics and outcomes of each PGT cycle as well as outcomes of the overall PGT procedure (number of cycles started, number of transfers, number of pregnancies achieved and their outcomes). COH outcomes were assessed by the total dose of exogenous gonadotropin, duration of ovarian stimulation, maximal E2 at the end of COH and the total number of retrieved oocytes. PGT-M outcomes were evaluated by the number of biopsied, diagnosed and unaffected embryos. Clinical pregnancy was defined by the presence of a fetal heartbeat at 6–7 weeks of pregnancy. All data were retrospectively retrieved from the electronic database (MEDIFIRST® software).

Throughout this manuscript, outcomes are primarily reported per couple, unless specified otherwise. Data referring to ovarian stimulation or genetic status are reported per patient (woman or man), while embryo-related outcomes are reported per embryo.

### Statistical analysis

The results are presented as mean ± SD or median and interquartile range (25–75%) for non-normal distributions and the number and percentage (%) for categorical variables. For the case–control analysis, COH outcomes were compared between cycles from women with sickle cell trait (AS) and matched control cycles (1:2 ratio) using univariate conditional logistic regression models to account for the matched design. For this analysis, continuous variables that did not follow a normal distribution were transformed into continuous variables (logarithmic transformation for BMI; square root for estradiol) or into categorical variables (number of stimulation days).

All statistical tests were two-sided, and a *P*-value of < 0.05 was considered statistically significant and were performed using NCSS Statistical Software (2021; NCSS 2021 Statistical Software, 2021, n.d.).

### Ethical approval

Consent for the use of their medical data for research was obtained from all couples at the time of the PGT-M attempt. Our database was approved by the National Data Protection Authority (Commission Nationale de l’Informatique et des Liberte´s, CNIL no. 1217921). This project is part of “research not involving the human person” (*RNIPH Recherche n’impliquant pas la personne humaine*), research framed at the legislative level by the regulations on data protection. In France, this type of work can be carried out without the advice and/or the approval of an ethics committee.

## Results

### Couple’s characteristics

**Fig. 1:**
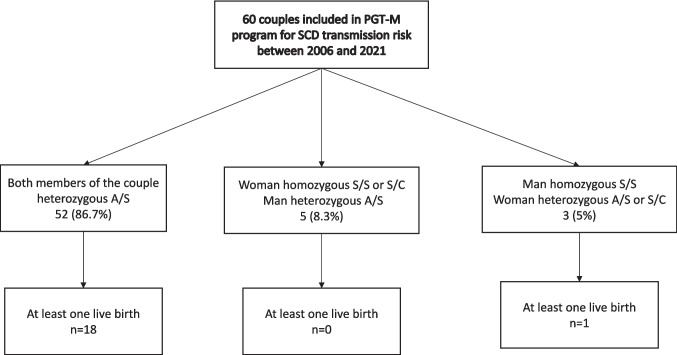
Flowchart of all couples in PGT-M program for SCD transmission risk between 2006 and 2021

In total, 60 couples were treated under the PGT-M program for SCD transmission risk between 2006 and 2021 at our PGT center (Fig. 1). The population description and the characteristics of the couples at the start of PGT-M procedure are described in Table 1. For 8 couples (13.3%), one partner suffered from SCD (SS or SC Genotype) (5 women and 3 men), so that the theoretical transmission risk of SCD was 50%. The majority of women (*n* = 48; 80%) were of African origin. Before the PGT procedure, 35 couples (58.3%) had at least one child affected by SCD (SS), and 17 of them (48.5%) requested PGT with HLA typing. A total of 19 couples (32%) had previously undergone a pregnancy termination for SCD prior to starting the PGT-M treatment. At the time of the first stimulation, the median age of women was 33 [31–35] years *versus* 39 [33–40] years for the men.

**Table 1:** Description of the population and characteristics at the time of the first PGD-M attempt (*n* = 60)

Patients characteristics	*n* (%) or median [25th–75th]
Women origin	
- Africa	48 (80.0)
- West Indies	11 (18.0)
- Asia	1 (2.0)
Sickle cells disease status of the couple	
- Both heterozygous	52 (86.7)
- Woman homozygous	5 (8.3)
- Man homozygous	3 (5.0)
Fertility status (*n* = 52)	
- No infertility	42 (80.7)
- Primary infertility	5 (9.6)
- Secondary infertility	5 (9.6)
At least one child affected with SCD S/S	35 (58.3)
At least one termination of pregnancy for SCD	19 (31.7)
Number of couples seeking PGT for SCD with HLA typing	17 (28.3)
Women age at (years)	33 [31–35]
Men age (years)	39 [33–40]
Women BMI (kg/m^2^)	26.2 [22.8–28.3]
Men BMI (kg/m^2^)	25.7 [22.5–27.5]
Ovarian reserve parameters at the first PGT-M attempt	
- AMH level (ng/ml)	3.5 [2.1–5.7]
- AFC	18 [13–23]

*SCD* sickle cell disease, *PGT-M* preimplantation genetic testing for monogenic disease

### Outcomes of the entire PGT program

The outcomes of the couples’ journey through PGT-M for SCD are detailed in Table 2 and Supplemental Table 1. During their PGT process, more than half of the couples (*n* = 34; 56.7%) underwent at least two ovarian stimulation cycles, while 45 couples (75.0%) had at least one fresh embryo transfer. A total of 21 frozen embryo transfers were performed for 13 couples (21.6.%). Four couples (6.7%) never had oocyte retrieval, and 11 couples (18%) were unable to have either a fresh or frozen embryo transfer. A total of 505 embryos were biopsied, and a diagnosis was obtained for 474 of them (93.9%). Among the diagnosed embryos, 261 (55.1%) were genetically unaffected, of which 172 (65.9%) were suitable for fresh or frozen embryo transfer. Detailed embryos results according to transmission risk are presented in Table 3.

**Table 2:** Flow of IVF–PGT-M cycles and outcomes according to sickle cell status

Outcome	Total	At-risk couples with no affected partner	At-risk couples with one affected partner
**IVF–PGT-M initiated cycles**	**123**	**103**	**20**
*Couple*	*60*	*52*	*8*
**Cycles with oocyte retrieval**	**108**	**90**	**18**
*Couple*	*56*	*48*	*8*
**Cycles with at least embryo biopsy**	**98**	**81**	**17**
Couple	*54*	*46*	*8*
**Cycles with ≥ 1 unaffected embryo**	**87**	**81**	**13**
*Couple*	*51*	*44*	*7*
**Cycles with at least one fresh embryo transfer**	**73**	**62**	**11**
*Couple*	*45*	*39*	*7*
**Live births after fresh transfer**	**15**	**14**	**1**
*Couple*	*15*	*14*	*1*
**Frozen embryo transfers**	**21**	**20**	**1**
*Couple*	*13*	*12*	*1*
**Live births after frozen transfer**	**4**	**4**	**-**
*Couple*	*4*	*4*	

Cycle-based and couple-based outcomes are presented separately to avoid ambiguity in denominators. Couples with sickle cell trait include those in which both partners were heterozygous carriers

**Table 3:** Embryo’s outcomes according to the transmission risk

	At-risk couples with no affected partner (*n* = 52)	Couples with affected woman partner (*n* = 5)	Couples with affected man partner (*n* = 3)
Total number of biopsied embryos	432	32	41
PGT-M results			
Diagnosed embryos	405/432 (93.8)	31/32 (96.9)	38/41 (92.7)
Undiagnosed embryos	27/432 (6.2)	1/32 (3.1)	3/41 (7.3)
Discordant embryos	41/405 (10.1)	6/31(18.8)	3/38 (7.9)
Affected embryos	125 (30.9)	16/31 (51.6)	22/38 (5.8)
Unaffected embryos	239 (59.0)	9/31 (29.0)	13/38 (34.2)
Outcome of unaffected embryos			
Embryos not suitable for transfer	82/239 (34.3)	3/9 (33.3)	4/13 (30.8)
Fresh embryo transfer	101/239 (42.2)	5/9 (55.6)	9/13 (69.2)
Frozen embryos	56/239 (23.4)	1/9 (11.1)	0 (0.0)

At the end of the PGT-M process, 19 couples (31.7%) had at least one live birth, including 4 couples who underwent PGT-M for SCD and HLA typing, of whom one had an HLA-unmatched embryo. Of the 19 couples who had a live birth, 15 achieved this after a fresh embryo transfer and the remaining four after a frozen embryo transfer.

#### PGT-M outcomes for affected women

For five couples in the PGT program, the woman was affected by SCD, four of them having experienced at least one pregnancy prior to entering the program, and three already had one child. One woman had SC genotype, while the remaining four were SS. Two women presented with renal involvement and one had ophthalmic complications. None of the five women were receiving hydroxyurea at the time of the protocol, had previously undergone HSCT, or had received exchange transfusions prior to inclusion. All of these women were evaluated and cleared by their hematologists to become pregnant. They also benefited from personalized management protocols adapted to their clinical condition before COH. These protocols were developed in collaboration with hematologists and anesthesiologists and included specific preventive measures such as systematic hydration protocols and thromboembolism prophylaxis. Over the course of the program, a total of nine stimulated cycles with oocyte retrievals were performed across the five couples with SCD affected women, resulting in a total of 32 biopsied embryos (Tables 2 and 3). A conclusive diagnosis was available for 31/32 embryos and nine (29.0%) were diagnosed as genetically unaffected, for four out the five couples. Ultimately, six unaffected embryos were transferred for these four couples (five after fresh embryo transfer and one after frozen embryo transfer). The remaining three unaffected embryos were neither transferred nor vitrified.

However, none of these women achieved a pregnancy or live birth following the PGT program. Importantly, none of the women experienced VOC, ACS, or other SCD-related complications during ovarian stimulation.

#### PGT-M outcomes for affected men

A total of three couples in the PGT program included a man affected by SCD; all were SS. Only one of the three men was receiving hydroxyurea treatment at the time of the procedure. He had cryopreserved sperm samples prior to treatment initiation which were used for fertilization due to subsequent azoospermia. The two other male partners had semen parameters compatible with ICSI, allowing the use of fresh sperm for fertilization. Across these three couples, nine stimulated cycles with oocyte retrievals were performed, resulting in 41 biopsied embryos. Of these, 13 embryos (31.7%) were genetically unaffected and transferred, either as fresh or frozen-thawed embryos and one live birth occurred (for the couple for whom cryopreserved sperm had been used) (Tables 2 and 3).

#### PGT-M outcomes for couples seeking for PGT for SCD and HLA typing

In the subgroup of the 17 couples seeking PGT for SCD with HLA typing, 33 ovarian stimulation cycles were initiated, and HLA-matched embryos were available in 11 cycles. Eleven fresh embryo transfers were performed in these couples, leading to 2 live births. Another live birth was achieved after a frozen embryo transfer. Overall, 3 HLA-matched births were obtained for the 17 requesting couples (17.6%). When a PGT-M cycle resulted in embryos that were healthy for SCD but not HLA-matched, the couples could choose whether or not to proceed with the transfer. As a result, a total of 11 embryo transfers were performed with embryos healthy for SCD but not HLA-matched, while 2 transfers were declined by couples due to HLA incompatibility. Thus, one couple had a healthy child but not HLA-matched.

### Ovarian stimulation characteristics and outcomes

A total of 123 ovarian stimulation cycles were initiated for the 60 couples included in the study. The characteristics and outcomes of these cycles are detailed in Table 4. The majority of ovarian stimulation cycles were performed using a programmed antagonist protocol pretreated with oral contraception (*n* = 62, 50.4%). Of the total cycles, 15 (12.2%) were canceled, with 14 due to insufficient ovarian response and one for personal reason.

**Table 4:** Ovarian stimulation and PGT-M outcomes

Characteristics of women at the start of stimulation (*n* = 123)	*n* (%) or med [25th–75th]
Cycles for sickle cell PGT/HLA matched, *n* (%)	29 (23.6)
Age of patients at stimulation (years)	34 [31–36]
AFC (follicles)	19 [14–23]
Protocol, *n* (%)	
- Pill/antagonist	62 (50.4)
- E2/antagonist	11 (8.9)
- Long delay/agonist	22 (17.9)
- Long/daily agonist	28 (22.8)
Initial dose of gonadotropins (IU)	300 [225–300]
Results of stimulation cycles (cycles with egg retrieval, *n* = 108)	
Total combined gonadotropin dose (IU)	2700 [1921–3300]
Treatment duration (days)	10 [9–12]
Parameter at trigger	
- E2 (pmol/l)	2770 [1956–3468]
- Progesterone (ng/ml)	0.58 [0.40–0.88]
- Number of follicles between 12 and 15 mm	5 [2–7]
- Dominant follicles (> 16 mm)	8 [8–16]
- Endometrium (mm)	10 [8–11]
Number of oocytes retrieved	11 [8–16]
Number of mature oocytes	10 [6–13]
Fertilization rate	72.7 [51.0–88.9]
Total number of embryos	7 [4–9]
Number of cycles with embryo biopsy	98 (90.7)
Results of cycles with embryo biopsies (*n* = 98)	
Number of biopsied embryos	4 [3–7]
Number of healthy embryos	2 [1–4]
Day of biopsy, n(%)	
- Day 3	75 (76.5)
- Day 4	19 (19.4)
- Day 5	4 (4.1)
Cycles with fresh embryo transfer (*n* = 73)	
Number of embryo transferred	
- SET (single embryo transfer)	30 (41)
- DET (double embryo transfer)	43 (59)
Day of embryo transfer	
- Day 4	23 (32)
- Day 5	50 (68)
Pregnancy rate	21 (28.7)
Outcome of the attempt	
- Ectopic pregnancy	1 (1.4)
- Early miscarriage	4 (5.5)
- Late miscarriage	1 (1.4)
- Live birth	15 (20.5)

Among the 108 remaining cycles that led to oocyte retrieval, the median total gonadotropin dose was 2700 [1921–3300] IU, administered over a treatment period of 10 [9–12] days. Oocyte retrieval yielded a median of 11 [8–16] oocytes, 80% being mature. Embryo biopsy was successfully performed in 98 cycles (90.7%) for 54 couples, with a median of 4 [3–7] embryos biopsied per cycle. In 87 cycles (88.8%), at least one healthy embryo was obtained (for 52 couples). Fresh embryo transfers were performed in 73 cycles (for 45 couples) resulting in 15 live births (20.5%). Two twin pregnancies (3%) were reported, representing 12% of all live births after fresh embryo transfer.

To assess the impact of sickle cell trait on ovarian stimulation response, we conducted a case–control study. Each cycle from a woman at risk of transmitting SCD was matched with two control cycles from women whose partners carried a chromosomal rearrangement. As only five women in the cohort had SCD, they were excluded to avoid bias, and the analysis was restricted to heterozygous women (sickle cell trait). In total, 99 cycles from heterozygous women were compared to 198 matched control cycles. Conditional logistic regression showed no significant differences in ovarian stimulation parameters between the two groups (Table 5).

**Table 5:** Comparison of the response to stimulation in heterozygous women and controls (partner of males with chromosomal rearrangement)

	Heterozygous women	Controls	*p*
Number of cycles	99	198	
Total dose of gonadotropins (IU)	2625 [1912–3300]	2542 [1912–3150]	0.13
Duration of treatment (days)	11 [9–12]	10 [8–11]	0.45
E2 on the day of trigger (pmol/L)	2783 [2030–3522]	2319 [1728–3175]	0.20
Number of oocytes retrieved	11 [8–16]	11 [8–15]	0.61
Number of mature oocytes	10 [6–13]	9 [6–13]	0.89

## Discussion

Our study aimed to analyze the outcomes of PGT-M for SCD risk in a single French center and to evaluate ovarian stimulation responses in women at risk of transmitting SCD. The theoretical risk of transmitting SCD ranges from 25 to 50%, depending on whether one partner is homozygous for the condition. In our study, 31.7% of the couples at risk of transmitting SCD had at least one live birth at the end of the PGT-M process.

Very few data are available on the outcomes of this strategy for this specific indication. Since the first study reporting the birth of twins following PGT-M for SCD published by Xu et al. in 1999 [8], only two studies have specifically reported PGT-M results for this indication [9, 10]. In 2009, Oyawo et al. reported the outcomes for 12 couples undergoing PGT for SCD. The live birth rate was 13% per initiated cycle, 18% per embryo transfer, and 17% per couple. In 2020, Vali et al*.* published a five-year experience with PGT-M for SCD risk. In this prospective single-center observational study involving 60 couples (52 with heterozygous carriers and 8 with one partner affected), 38 couples achieved a live birth, corresponding to a cumulative live birth rate of 63% per couple. In our study, including the same number of couples included in PGT-M program for SDC risk transmission, the cumulative live birth rate per couple was 31.7%. This difference in outcomes is challenging to explain, as the general characteristics of the patients, transmission risks, protocols used and percentage of healthy embryos were relatively similar in both studies. However, the cumulative live birth rate observed in our study remains within the range reported in France for IVF with PGT-M of monogenic indications (approximately 27–45%) [13–15]. Comparisons of live birth rates across studies should be interpreted with caution due to small cohort sizes involved and methodological differences, including criteria for patient selection, the timing of biopsy (day-3 versus day-5 embryos) and embryo transfer strategies (fresh and frozen transfers versus exclusively frozen transfers). Moreover, a complementary analysis based on the period of inclusion suggested an improvement in outcomes over time, with higher cumulative live birth rates among couples initiating PGT-M from 2015 onward (43.5%, 10/23) compared with those initiating treatment before 2015 (24.3%, 9/37). This trend may reflect advances in blastocyst-stage biopsy, cryopreservation techniques, and IVF laboratory practices.

If PGT-M results improve, there will be a greater temptation to have access to PGT. Interestingly, Combs et al. assess the cost-effectiveness of PGT for SCD [16], and compared PGT with other reproductive options, such as prenatal diagnosis (PND) and pregnancy termination. Their results suggest that while PGT can be more costly upfront, it may be a cost-effective option in preventing the birth of children with SCD, particularly when considering the long-term healthcare costs of managing SCD and the fact that, in the Afro-Caribbean population, pregnancy termination is seldom chosen. Among the 60 couples included in the process, 19 (32%) had undergone pregnancy termination following PND before considering PGT-M. However, we lack data on the number of couples who did not proceed to PND or chose to continue pregnancies despite PND revealing an affected fetus. PND and potential pregnancy termination are often experienced as highly traumatic, making PGT-M a valuable alternative to avoid such distress. Additionally, over 50% (*n* = 35) of the couples in our cohort already had one or more children affected by SCD. The challenges faced by these children likely influenced the couples’ decision to pursue PGT-M for SCD prevention. It has been shown that couples who could benefit from PGT-M for SCD risk are often unaware of the possibility of accessing this option, even though they would be willing to use it [17–19]. Thus, there is a crucial lack of information available to couples who could benefit from PGT-M, and educational tool to inform patients about PGT should be developed. This lack of information has recently been highlighted by studies evaluating patient education in the context of sickle cell disease, showing that tailored educational tools are well accepted and improve understanding of reproductive options [19]. In contrast, standard PGT educational materials frequently exceed national literacy recommendations, potentially limiting patient comprehension and informed decision-making [20]. The PGT-M process is relatively long, demanding, and generates anxiety, as there is a significant risk of failure at each stage of the procedure. For this reason, a consultation with a reproductive medicine specialist is essential to explain the process, protocols, potential complications, and chances of success before starting [21].

PGT for SCD can also serve a therapeutic purpose. To perform a hematopoietic stem cell transplant for a child with S/S sickle cell disease, parents may request HLA typing of healthy embryos to identify compatibility with an affected sibling [22]. In such cases, cells from the placenta or umbilical cord are collected at birth, and a bone marrow transplant can later be performed. In our study, 17 couples (28.3%) sought PGT with HLA compatibility testing, but only 3 HLA-matched births were achieved. Given these disappointing results, and ethical tensions due to the French law constraints that prohibit attempting a new stimulation cycle when healthy embryos are cryopreserved, this procedure is no longer performed in our center. These regulatory limitations raise ethical questions regarding access to HLA-matched PGT-M, particularly for families affected by severe chronic diseases. Whether such policies may contribute to healthcare disparities warrants further ethical and societal discussion beyond the scope of this study, but should take place as part of the next review of the French Bioethics Law scheduled for 2026.

As a study reported a possible increased of vascular complication as preeclampsia in heterozygous women [23], we explored the response to COH in women presented with sickle cell trait as compared to controls. We found no significant difference in stimulation response between the controls and the women at risk of transmitting the disease. These findings are reassuring regarding the possibility of performing IVF-ICSI with PGT-M in this context. However, it should be emphasized that women eligible for PGT-M were selected based on ovarian reserve parameters, at the time of the PGT-M demand (approximately 18–24 months before the first ovarian stimulation cycle), which introduces a selection bias. As a consequence,, we cannot draw conclusions about the impact of a heterozygous mutation on ovarian reserve. Moreover, as the vast majority of our patients were heterozygous, it is not possible to extrapolate our results to women affected by SCD. In our study, only 5 women were affected by SCD. Due to this low number of homozygous women in our cohort, we are unable to draw conclusion concerning ovarian reserve, COH and PGT-M outcomes in this population. Several studies suggest a potential impairment of fertility in SCD patients, which may have a iatrogenic origin (repeated transfusions, hydroxyurea treatment, hematopoietic stem cell transplantation), or result from hypogonadotropic hypogonadism or alteration of ovarian reserve [24, 25]. Since women were selected based on ovarian reserve parameters to access the PGT program, we cannot assess the impact of the disease on ovarian reserve. Moreover, if none of the affected women had a pregnancy through PGT in our center, four of them had already experienced at least one spontaneous pregnancy prior to PGT, indicating that they did not present with infertility. At least, some studies have reported an increased risk of VOC, ACS, and thromboembolic disease during ovarian stimulation for fertility preservation or infertility treatment outside of PGT-M [26]. In our series, none of the women experienced VOC, ACS, or other SCD-related complications during ovarian stimulation. This absence of complications may be explained, by the selection of patients whose clinical condition was compatible with pregnancy, and by the implementation of individualized preventive strategies developed in collaboration with hematologists and anesthesiologists. These protocols were designed to address the increased risks associated with ovarian stimulation and to ensure the safety of the patients throughout the process. This raises the question of whether systematic thromboprophylaxis should be implemented for this population in association with adapted measures and monitoring. Unfortunately, we did not assess the obstetric and neonatal complications of pregnancies resulting from PGT-M.

## Conclusion

In conclusion, PGT-M for SCD is a procedure that has enabled the birth of a healthy child in approximately 30% of cases in our center. The ovarian stimulation response in women at risk to transmitting SCD seems not significantly different from that of a control group. These findings are crucial for providing couples considering PGT with comprehensive information. It is important to weigh the benefits and risks of such a procedure, which include preventing the birth of an affected child and avoiding the risk of pregnancy termination, but also the potential risks associated with ovarian stimulation and IVF in SCD patients. Our study aims to assist geneticists, gynecologists, and hematologists in providing the necessary guidance before embarking couples on this long and often challenging journey.

## Supplementary Information

Below is the link to the electronic supplementary material.

Supplementary file1 (DOCX 14.8 KB)

## Data Availability

The data underlying this article will be shared on reasonable request to the corresponding author.
