# Supplementary material for: Effectiveness of preimplantation genetic testing in sickle cell disease: insights from a single-center experience
Source: J Assist Reprod Genet. 2026 Feb 5;43(4):1233–42. doi: 10.1007/s10815-026-03809-1 (PMC13103162; doi:10.1007/s10815-026-03809-1)
Supplement: Supplementary file 1 — Supplementary file1 (DOCX 14.8 KB) [file 10815_2026_3809_MOESM1_ESM.docx]

Supplemental table 1. Characteristics of couple’s courses of PGT-M for sickle cell disease (n=60 couples)

|  | n (%) |
| --- | --- |
| Total number of IVF/PGT-M cycles started per couple  - 1  - 2  - ≥ 3 | 26 (43.3)  16 (26.7)  18 (30.0) |
| Total number of egg retrievals per couple  - 0  - 1  - 2  - ≥ 3 | 4 (6.7)  27 (45.0)  15 (25.0)  14 (23.3) |
| Total number of fresh embryo transfers per couple  - 0  - 1  - 2  - ≥ 3 | 15 (25.0)  26 (43.3)  12 (20.0)  7 (11.7) |
